# Adverse Reactions Due to Directly Observed Treatment Strategy Therapy in Chinese Tuberculosis Patients: A Prospective Study

**DOI:** 10.1371/journal.pone.0065037

**Published:** 2013-06-04

**Authors:** Xiaozhen Lv, Shaowen Tang, Yinyin Xia, Xiaomeng Wang, Yanli Yuan, Daiyu Hu, Feiying Liu, Shanshan Wu, Yuan Zhang, Zhirong Yang, Dehua Tu, Yixin Chen, Peiyuan Deng, Yu Ma, Ru Chen, Siyan Zhan

**Affiliations:** 1 Department of Epidemiology and Biostatistics, School of Public Health, Peking University Health Science Centre, Beijing, China; 2 Clinical Research Division, Peking University Institute of Mental Health, and Key Laboratory for Mental Health, Ministry of Health, Beijing, China; 3 Department of Epidemiology and Biostatistics, School of Public Health, Nanjing Medical University, Nanjing, China; 4 National Center for Tuberculosis Control and Prevention, Chinese Center for Disease Control and Prevention, Beijing, China; 5 Center for Disease Control and Prevention in Zhejiang Province, Hangzhou, China; 6 Center for Disease Control and Prevention in Jilin Province, Changchun, China; 7 Center for Disease Control and Prevention in Chongqing Municipality, Chongqing, China; 8 Center for Disease Control and Prevention in Guangxi Zhuang Autonomous Region, Nanning, China; 9 Beijing Institute for Tuberculosis Control, Beijing, China; 10 Center for Drug Reassessment, State Food and Drug Administration, Beijing, China; 11 Beijing Center for Drug Reassessment, Beijing, China; 12 Beijing Tuberculosis and Thoracic Tumor Research Institute, Beijing, China; Institute of Infectious Diseases and Molecular Medicine, South Africa

## Abstract

**Background:**

More than 1 million tuberculosis (TB) patients are receiving directly observed treatment strategy (DOTS) therapy in China every year. As to the profile of adverse drug reactions (ADRs) due to DOTS therapy, no consensus has been reached. There is no report regarding ADRs due to DOTS therapy with a large Chinese TB population. This study aimed to determine the incidence and prognosis of ADRs due to DOTS therapy, and to evaluate their impact on anti-TB treatment in China.

**Methods:**

A prospective population-based cohort study was performed during 2007–2008. Sputum smear positive pulmonary TB patients who received DOTS therapy were included and followed up for six to nine months in 52 counties of four regions in China. The suspected ADRs were recorded and reviewed by Chinese State Food and Drug Administration.

**Results:**

A total of 4304 TB patients were included in this study. 649 patients (15.08%) showed at least one ADR and 766 cases in total were detected. The incidence (count) of ADR based on affected organ was: liver dysfunction 6.34% (273), gastrointestinal disorders 3.74% (161), arthralgia 2.51% (108), allergic reactions 2.35% (101), neurological system disorders 2.04% (88), renal impairment 0.07% (3) and others 0.05% (2). Most cases of ADRs (95%) had a good clinical outcome, while two with hepatotoxicity and one with renal impairment died. Compared with patients without ADRs, patients with ADRs were more likely to have positive smear test results at the end of the intensive phase (adjusted OR, 2.00; 95%CI, 1.44–2.78) and unsuccessful anti-TB outcomes (adjusted OR, 2.58; 95%CI, 1.43–4.68).

**Conclusions:**

The incidence of ADRs due to DOTS therapy was 15.08%. Those ADRs had a substantial impact on TB control in China. This highlighted the importance of developing strategies to ameliorate ADRs both to improve the quality of patient care and to control TB safely.

## Introduction

Tuberculosis (TB) continues to be a major cause of morbidity and mortality worldwide, with 8.7 million new cases of TB and 1.4 million people died from TB globally in 2011 [1]. China ranked second amongst all TB high-burden countries, having 1.0 million incident cases and 47,000 deaths in 2011 [1]. In order to control the TB epidemic, China established China National Tuberculosis Prevention and Control Scheme in 1990 and has implemented directly observed treatment strategy (DOTS) therapy since 1991, which is the cornerstone of the current strategy for TB control and covers the entire population of China [2], [3]. The key component of DOTS therapy is the standard anti-TB short course chemotherapy regimen, which requires continually taking drug combinations of isoniazid (INH), rifampicin (RFP), pyrazinamide (PZA), ethambutol (EMB) and/or streptomycin (SM) every other day for six to nine months [4].

Despite the positive therapeutic effects, studies have shown that utilization of multidrug regimens can cause undesirable adverse drug reactions (ADRs) of varying degrees of severity, such as hepatotoxicity, gastrointestinal disorders, allergic reactions, arthralgia, neurological disorders and so on [5]–[16]. ADRs increase patient suffering and incur substantial additional costs because of added outpatient visits, tests, and in more serious instances hospitalizations [13], [17]. In addition, ADRs are regarded as one of the major causes of non-adherence to anti-TB treatment [18]. At the same time, alternative agents may have greater problems with toxicity, and are often less effective. As a result, ADRs may eventually contribute to the extension of treatment duration, final termination, drug resistance and treatment failure [19]. It may also increase the number of TB cases, and more rarely the number of deaths, posing a challenge to the management of TB patients and TB control.

The frequency, severity and the nature of anti-TB therapy induced ADRs have always been a matter of concern [7]. As to the overall incidence of ADRs caused by anti-TB therapy, no consensus has been reached worldwide, with the incidence of ADRs ranging from 5.1% to 83.5% [5]–[8], [10]–[16], [20]. Most individual studies of Chinese patients in our previous systematic review had a small sample size, different definitions of ADRs and were done in hospitals, where limitations existed to reflect the profile of ADRs due to DOTS therapy in China [21]. To our knowledge, there is no report regarding ADRs due to DOTS therapy with a large Chinese TB population and minimal epidemiologic data exists concerning the impact of ADRs on anti-TB treatment.

In this study, we aimed to get an overview of ADRs due to DOTS therapy and evaluate their impact on anti-TB treatment with a large population-based prospective study in China.

## Methods

### Ethics Statement

The prospective study was approved by the Ethics Committee of Center for Tuberculosis Control and Prevention of China. Written informed consent was obtained from every participant or surrogate before enrolment.

### Patient’s Enrollment

A prospective cohort study was performed in Anti-tuberculosis Drugs Induced Adverse Reactions in China National Tuberculosis Prevention and Control Scheme Study (ADACS) from October 2007 to June 2008 [2]. All selected regions performed DOTS therapy and represented diverse TB patients in China. Four geographically and economically diverse regions of China were selected with expert consultancy. A total of 52 out of 299 counties were randomly selected from all eligible counties in the four regions. In each county the number of participants to be sampled was decided using the probability proportional to size sampling strategy, in accordance with the proportion of the counties’ new reported sputum smear-positive TB patients in 2006. All sputum smear positive pulmonary TB patients who accepted DOTS therapy were potential eligible participants. Patients with any of the following conditions were excluded from ADACS: ? a psychiatric disease that led to unable to adequately fill in the questionnaires,including baseline questionnaire, following up form for recording patients’ medication, ADRs, anti-TB outcomes and calendars for self-recording any signs or symptoms of ADRs; ? a serious diseases with a prognosis shorter than six months; ? problems with signing a consent form. TB patients participated in ADACS program voluntarily. During the recruitment phase, 6,460 smear-positive patients who received DOTS therapy were identified, 155 patients did not meet the study inclusion criteria and 1,817 patients did not respond to the study. Therefore, a total of 4,488 patients were recruited. There were no significant differences in age and gender distribution between the 4488 participants and the smear positive patients not included. All primary/re-treatment patients received oral INH (600 mg), RFP (600 mg, or 450 mg if body weight was <50 kg), PZA (2000 mg), and EMB (1250 mg) every other day for the first two months (intensive phase) and INH and RFP for another four to six months (consolidation phase). The re-treatment of patients meanwhile received SM (750 mg) every other day in the first two months and continued receiving EMB for another six months. All subjects should receive a smear test at the end of the intensive phase and at the end of anti-TB treatment. More details about ADACS can be found in our previous report [2].

### Investigation and Following Up

Before anti-TB therapy, participants recruited completed the baseline questionnaire and received several laboratory examinations, including blood routine test (the number of red blood cells, white blood cells and platelet and the level of hemoglobin in full blood count), urine routine test (PH value, the level of uric acid and urine protein, cylindruia, and the number of red blood cells and white blood cells in urine), liver and renal function test, hepatitis B surface antigen (HBsAg) test. During the follow up period, blood and urine routine test as well as liver and renal function test were measured again within two months after anti-TB treatment initiation. The average time for laboratory examinations performed after initiation of therapy was 47 days in this study. If suspected ADR symptoms arose, the same tests were administered again. The participants were instructed to use ADACS calendars to self-record any signs or symptoms of ADRs and to report to the local specialist clinicians if they had discomfort or adverse reactions. Local specialist clinicians offered monitoring for ADRs and checked the ADACS calendars regularly. Once a suspected ADR was identified, the clinicians recorded and followed up until resolution or end of TB therapy. ADR patients modified their DOTS therapy and/or received symptomatic therapy according to the seriousness of the ADR. Follow up was provided to all participants until the completion of DOTS therapy. Patients who either transferred out of the therapy (moving house/working outside leading to loss following up but having no ADRs before this), or died because of reasons other than ADRs during the monitoring were excluded from the present study.

### ADRs Definition

The suspected ADRs were then reported to Center for Drug Reassessment of Chinese State Food and Drug Administration for evaluation. ADR was defined as an appreciably harmful or unpleasant reaction, resulting from an intervention related to the use of a medicinal product, which predicts hazard from future administration and warrants prevention or specific treatment, or alteration of the dosage regimen, or withdrawal of the product [22]. The causality was evaluated following the standards of WHO Uppsala Monitoring Center System [23] by experts from Center for Drug Reassessment of Chinese State Food and Drug Administration. Considering safety and practical necessity, rechallenge with the suspected drug was not done for most cases. Therefore, ADRs designated in this study were those suspected ADRs with certain, probable or possible causality assessment results. Serious ADRs were defined as any untoward medical occurrence that at any dose results in death, requires hospital admission or prolongation of existing hospital stay, results in persistent or significant disability/incapacity, or is life threatening [22]. Liver dysfunction was accepted as an increase in serum alanine aminotransferase (ALT), aspartate aminotransferase (AST) or total bilirubin greater than two times the upper limit of normal (ULN) or higher than ULN in two continuous tests conducted in a two week interval, not considering the symptoms. Hepatotoxicity was defined as an increase in ALT or AST that was greater than three times of ULN, or in total bilirubin greater than two times of ULN [24]. Hyperuricemia was defined as an increase in uric acid levels of more than 8 mg/dl. Anemia was defined as hemoglobin (Hgb) concentration <11 g/dl in male and <10 g/dl in female in patients without a history of anemia or more than 1 g/dl drop in Hgb concentration after anti-TB treatment. Neutropenia and thrombocytopenia were recognized as a drop in absolute neutrophil count and platelet count equal to or less than 1500 cells/mm^3^ and less than 150000 cell/mm^3^ respectively. Except liver dysfunction, hematologic system disorders and renal impairment were determined based on laboratory examination, other ADRs including allergic reactions, arthralgia and nervous system disorders were determined based on symptoms. Nervous system disorders included auditory nerve damage, optic nerve damage, peripheral nervous damage and central nervous system damage.“Others” referred to those ADRs could not classified to above types, such as interstitial pneumonia, hyperthyroidism, lipsotrichia and so on.

### Assessment of Anti-TB Treatment Effect

In this study, smear results at the end of intensive phase were used as the interim index and anti-TB outcomes at the end of consolidation phase were used as the total index of anti-TB treatment effect. Anti-TB outcomes were classified into two categories: successful outcomes (defined as the completion of treatment and patients being cured) and unsuccessful outcomes, including treatment failure, default and death because of ADRs due to DOTS therapy [25].

### Statistical Methods

Results were expressed as median and interquartile range (IQR) or as numbers and percentages. Descriptive statistics were used to determine frequency and prognosis of each type of ADRs. The estimated incidence of ADRs was standardized for age and gender with direct standardization using one reference population from the national 2008 TB epidemic surveillance database [26].

The potential impact of ADRs on smear results at the end of intensive phase and anti-TB outcomes were assessed with odds ratio (OR), attributable risk proportion (AR%) and population attributable risk proportion (PAR%), respectively. The impact of ADRs on anti-TB treatment was also evaluated by estimating ORs adjusted for age, gender, TB treatment history and disease history/HBsAg status using multivariable Logistic regression analysis. The ADRs whose impact were evaluated on smear results at the end of intensive phase were only those who developed ADRs within the intensive phase, while on anti-TB outcomes were all the ADRs in the cohort. Statistical analysis was performed using SPSS for Windows (version 13.0, SPSS Inc.,).

## Results

### Characteristics of Patients

During the 10 month period, a total of 4488 TB patients were enrolled in this study. Among these patients, 129 dropped out during monitoring (owing to moving house and working outside), 23 patients died due to TB, and 32 died due to other reasons including heart disease, cancer and accidents. As a result, 4304 patients were included in this study, with a median observational time of 184 days. Compared with the 2008 national smear positive TB patients in China [26], the gender distribution of the subjects (male 71.6% and female 28.4%) was similar to the national 2008 TB epidemic surveillance database (male 71.5% and female 28.5%), while the proportion of patients older than 65 years old in this study (11.5%) was smaller than that in the national TB population (19.8%). Table 1 shows the baseline characteristics of 4304 TB patients.

**Table 1:** Baseline Characteristics of 4304 Chinese tuberculosis patients.

Parameter	Number(%)
Number of patients	4304
Age, years, median (IQR)	42(29–55)
Male/female	3082/1222(71.60/28.40)
Tuberculosis treatment history	
Primary	3556(82.62)
Re-treatment	748(17.38)
Education level	
None/elementary school	1895(44.03)
High school	2260(52.51)
College/higher	135(3.14)
Missing	14(0.32)
Weight, kg, median(IQR)	52.70(48.00–58.00)
Body mass index, kg/m^2^, median(IQR)	19.23(17.75–20.96)
HBsAg positive	469(10.89)
History of drug allergy/reaction	118(2.74)
History of disease	
Hepatic and bilinary disease	23(0.54)
Gastroenteropathy	40(0.93)
Nephropathy	17(0.40)
Diabetes	51(1.18)
Others	103(2.40)

IQR, inter-quartile range.

### Incidence and Prognosis of ADRs

The types, incidence, onset time and seriousness of ADRs due to DOTS therapy are listed in Table 2. A total of 649 patients showed at least one ADR (649/4304, 15.08%, standardized was 15.48%), including 550 patients with one ADR, 82 with two, 16 with three and one with four, with a total of 766 cases detected. Liver function disorder was the most common ADR (273, 6.34%) and 106 of those experienced hepatotoxicity (2.55%). The other frequent ADRs were gastrointestinal disorder, arthralgia, allergic reactions, and nervous system disorders. The incidences of the aforementioned ADRs were 3.74%, 2.51%, 2.35%, and 2.04%, respectively. Regarding the seriousness of ADRs, 92.30% ADRs were non-serious. A total of 518 patients with ADRs (79.82%) were identified during the intensive phase following initiation of anti-TB treatment. The median interval in days between the initiation of DOTS therapy and the detection of ADRs was 35 (IQR 14–59).

**Table 2:** Incidence, onset time and seriousness of adverse drug reactions due to directly observed treatment strategy therapy in 4304 Chinese tuberculosis patients.

Type	Number of patients (Incidence, %)	Standardized incidence#, %	Onset time†, days, median(IQR)	Seriousness, number of patients (%)	
				Non-serious	Serious§
Liver dysfunction	273(6.34)	6.21	53(28–60)$	249(91.21)	24(8.79)
Gastrointestinal disorders	161(3.74)	4.03	16(6–51)	148(91.93)	13(8.07)
Arthralgia	108(2.51)	2.57	54(28–59)	106(98.15)	2(1.85)
Allergic reactions	101(2.35)	2.47	20(6–46)	91(90.10)	10(9.90)
Nervous system disorders‡	88(2.04)	2.15	17(6–54)	83(94.32)	5(5.68)
Hematologic system disorders	30(0.70)	0.75	55(31–84)$	26(86.67)	4(13.33)
Renal impairment	3(0.07)	0.09	30(29–36)$	2(66.67)	1(33.37)
Others^€^	2(0.05)	0.06	missing	2(100.00)	0(0.00)
Total	766(17.33)*	–	35(14–59)	707(92.30)	59(7.70)

IQR, inter-quartile range.

# The incidence of ADR was standardized for age and gender with direct standardization using one reference population that from national TB epidemic surveillance database of 2008.

† It was from initiation of treatment.

§ Serious ADRs were defined as any untoward medical occurrence that at any dose results in death, requires hospital admission or prolongation of existing hospital stay, results in persistent or significant disability/incapacity, or is life threatening.

$ It was the time that ADRs were found, not the exact time it happened.

‡ Nervous system disorders included auditory nerve damage, optic nerve damage, peripheral nervous damage and central nervous system damage.

€ Others included one with interstitial pneumonia and another with hypokalemia.

* For 82 patients got two ADRs, sixteen got three and one got four, 766 cases were detected and the denominator was 4304+82+32+3 = 4421.

The causality assessment of ADRs results showed that 219 cases (28.6%) were certain and 407 (53.1%) were probable (Table 3). Symptomatic therapy was one of the major measures managing ADRs. A total of 593 (77.4%) patients with ADRs had extra clinic visits and 523 (68.3%) received treatment, including 42 (5.5%) who required hospitalization (Table 4). Most cases of ADRs (95%) had a good clinical outcome, but two patients with hepatotoxicity and one with renal impairment died of ADRs.

**Table 3:** Causality assessment of adverse drug reactions due to directly observed treatment strategy therapy*.

Type(number)	Certain#	Probable#	Possible#
Liver dysfunction(273)	33(12.1)	177(64.8)	63(23.1)
Gastrointestinal disorders(161)	71(44.1)	74(46.0)	16(9.9)
Allergic reactions(101)	43(42.6)	38(37.6)	20(19.8)
Arthralgia(108)	22(20.4)	72(66.6)	14(13.0)
Nervous system disorders(88)	34(38.6)	33(37.5)	21(23.9)
Hematologic system disorders(30)	15(50.0)	9(30.0)	6(20.0)
Renal impairment(3)	1(33.3)	2(66.6)	0(0.0)
Others(2)	0(0.0)	2(100.0)	0(0.0)
Total	219(28.6)	407(53.1)	140(18.3)

* The causality assessment was done following the standards of WHO Uppsala Monitoring Center System by the experts from Center for Drug Reassessment of Chinese State Food and Drug Administration.

# Data was presented as number of patients (%).

**Table 4:** Symptomatic therapy for adverse drug reactions due to directly observed treatment strategy therapy.

Type*	Total number	Clinic visit#	Examination^#$^	Therapy^#$^	Hospitalization#
Liver dysfunction	273	219(80.2)	195(71.4)	213(78.0)	20(7.3)
Gastrointestinal disorders	161	123(79.4)	48(31.8)	108(67.9)	7(4.9)
Allergic reactions	101	81(83.5)	31(33.7)	91(90.1)	10(11.0)
Arthralgia	108	83(79.8)	63(63.6)	50(47.6)	0(0.0)
Nervous system disorders	88	58(74.4)	16(21.9)	36(45.6)	3(4.2)
Hematologic system disorders	30	26(92.9)	25(92.6)	23(82.1)	2(7.7)
Renal impairment	3	3(100.0)	1(33.3)	2(66.7)	0(0.0)
Total	764	593(77.6)	379(49.6)	523(68.5)	42(5.5)

* The related data of other adverse drug reactions (2 cases) missed.

# Data was presented as number of patients (%).

§ Some patients had clinic visit just to consult and get some medicine, but had no examination. As a result, the number of having examination was smaller than that having clinic visit.

$ “Therapy” referred to symptomatic therapy for ADRs, such as liver protective drugs, drugs for alleviating gastrointestinal disorders, nervous system disorders, but not included changes in anti-TB therapy.

### Impact on Anti-TB Treatment of ADRs

As to the impact of ADRs on anti-TB treatment pattern, 325 (7.55%) patients required modifying their anti-TB treatment, including interruption, dose reduction, changes in medical administration (changing from taking medicines “every other day” to “every day” or “on an empty stomach” to “after a meal”), drug replacement or discontinuation. A total of 48.0%, 54.7% and 52.3%% of patients with liver dysfunction, gastrointestinal disorders and nervous system disorders respectively had to change their anti-TB treatment pattern. Compared with other ADRs, arthralgia had the least impact on anti-TB treatment pattern (13.0% changed). The details are shown in Table 5.

**Table 5:** Influence of adverse drug reactions due to directly observed treatment strategy therapy on anti-TB treatment pattern.

Type*	Total number	Anti-TB treatmentpattern changed#	Forms of anti-TB treatment pattern changed#				
			Interruption#	Dose reduction#	changes in medicaladministration^#&^	Drug replacement#	Discontinuation^#^ §
Liver dysfunction	273	131(48.0)	83(30.4)	6(2.2)	18(6.6)	32(11.7)	15(5.5)
Gastrointestinal disorders	161	88(54.7)	10(6.2)	11(6.8)	51(31.7)	15(9.3)	10(6.2)
Allergic reactions	101	36(35.6)	13(12.9)	2(2.0)	8(7.9)	13(12.9)	5(5.0)
Arthralgia	108	14(13.0)	3(2.8)	1(0.9)	5(4.6)	2(1.9)	2(1.9)
Nervous system disorders	88	46(52.3)	8(9.1)	0(0.0)	25(28.4)	9(10.2)	7(8.0)
Hematologic system disorders	30	10(33.3)	6(20.0)	2(6.7)	0(0.0)	5(16.7)	1(3.3)
Total	761	325(42.7)	123(16.2)	22(2.9)	107(14.1)	76(10.0)	40(5.3)

TB, tuberculosis.

* The related data of renal impairment and other adverse drug reactions (5 cases) missed.

# Data was presented as number of patients (%).

& Changes in medical administration included taking medicines “every other day” changing to “every day” or “on an empty stomach” changing to “after a meal”.

§ Patients with ADRs refused to continuing anti-TB treatment for worrying about ADRs or loss patience.

At the end of intensive phase, a total of 4253 patients (4253/4304, 98.8%) had smear tests performed and 4015 (94.4%) were negative and 238 (5.6%) positive. At the end of consolidation phase, 4288 patients’ (4288/4304, 99.6%) anti-TB outcomes were assessed and 4234 (98.7%) were successful and 54 (1.3%) unsuccessful. The possible risk factors except ADRs for the smear results at the end of intensive phase and anti-TB outcomes at the end of consolidation phase were shown respectively in Table 6. Before and after adjusting for age, gender, TB treatment history and disease history/HBsAg status, ADRs both had substantial negative impact on smear results at the end of intensive phase and anti-TB outcomes. Compared with patients without ADRs in our cohort, patients with ADRs were more likely to have positive smear results at the end of the intensive phase (adjusted OR, 2.00; 95%CI, 1.44–2.78). Patients with ADRs were also more likely to develop unsuccessful outcomes (adjusted OR, 2.58; 95%CI, 1.43–4.68). PAR% for positive smear results at the end of the intensive phase and unsuccessful outcomes attributed to ADRs in TB patients was 10.75% (95% CI, 9.82%–11.67%) and 19.22% (95% CI, 18.04%–20.40%) respectively. Thus approximately 10.75% of all patients with positive smear result at the end of the intensive phase and 19.22% of all patients with unsuccessful outcomes may be attributed to ADRs due to DOTS therapy (Table 7).

**Table 6:** Possible risk factors for the smear results at the end of intensive phase and anti- tuberculosis outcomes at the end of consolidation phase, respectively.

Parameter	Smear test results*				Anti-TB outcomes#			
	N	Positive	Negative	*P* value	N	Unsuccessful	Successful	*P* value
Age, years, median (IQR)	4253	44(34–58)	41(28–55)	0.003	4288	49(39–59)	41(29–55)	0.004
Male/female	4253	179/59	2867/1148	0.206	4288	43/11	3026/1208	0.186
TB treatment history	4253			0.002	4288			0.189
Primary		179	3337			41	3503	
Re-treatment		59	678			13	731	
Education level	4239			0.164	4274			0.191
None/elementary school		116	1748			29	1855	
High school		117	2127			25	2230	
College/higher		4	127			0	135	
Weight, kg, median(IQR)	4246	52(47–58)	53(48–58)	0.108	4281	52(45–60)	53(48–58)	0.659
HBsAg Status	4037			0.637	4067			<0.0001
Positive		28	431			15	450	
Negative		199	3379			36	3566	
History of drug allergy/reaction	4226			0.524	4258			0.708
Yes		8	108			1	117	
No		227	3883			51	4089	
History of disease	4239			0.001	4272			0.573
Yes		24	208			2	230	
No		214	3793			52	3988	

IQR, inter-quartile range.

* For the data of 51 patients missed, 4253 patients’ data were analyzed.

# For the data of 16 patients missed, 4288 patients’ data were analyzed. Successful outcomes defined as the completion of treatment and patients being cured. Unsuccessful outcomes defined as treatment failure, default and death because of ADRs due to DOTS therapy.

**Table 7:** Impact of ADRs due to directly observed treatment strategy therapy on smear results at the end of intensive phase and anti-TB treatment outcomes.

Category	Patientswith ADRs	Patients without ADRs	OR (95% CI)	Adjusted OR(95% CI)	AR%# (95% CI)	PAR%# (95% CI)
Smear results at the end ofintensive phase^ &^						
Positive	50	188	2.11(1.52–2.92)	2.00*(1.44–2.78)	50.00(48.51–51.49)	10.75(9.82–11.67)
Negative	450	3565				
Anti-TB treatment outcomes$						
Unsuccessful	17	37	2.70(1.51–4.82)	2.58§(1.43–4.68)	61.24(59.78–62.70)	19.22(18.04–20.40)
Successful	616	3618				

ADRs, adverse drug reactions; TB, tuberculosis; OR, odds ratio; CI, confidence internal; AR%, attributable risk proportion; PAR%, population attributable risk proportion.

& A total of 518 patients developed ADRs within the intensive phase. For the data of 51 patients missed, 4253 patients’ data were analyzed.

$ A total of 649 patients developed ADRs at the end of anti-TB treatment. For the data of 16 patients missing, 4288 patients’ data was analyzed. Successful outcomes defined as the completion of treatment and patients being cured. Unsuccessful outcomes defined as treatment failure, default and death because of ADRs due to DOTS therapy.

* Age, gender, TB treatment history and disease history were adjusted using logistic regression analysis.

§ Age, gender, TB treatment history and HBsAg status were adjusted using logistic regression analysis.

# AR% and PAR% were calculated based on adjusted OR, respectively.

## Discussion

### Incidence and Prognosis of ADRs

Anti-TB drugs could cause significant adverse effects both in quantity and severity [6]. The results of this study indicated that ADRs due to DOTS therapy is a problem which should be highlighted. In this study, among 4304 active TB patients, 649 patients (15.03%) experienced at least one ADR and 766 cases in total were detected. Liver dysfunction was the most common ADR (6.34%), which accounted for the largest proportion (35.64%) of all ADRs due to DOTS therapy. The incidence of hepatotoxicity was 2.55%, with more details found in our previous report [24]. The other frequent ADRs were gastrointestinal disorder, arthralgia, allergic reactions, and nervous system disorders. The order of the ADR incidence was similar to other studies [6], [13]. Compared with most similar studies [6], [7], [10], [11], [13], [15], the incidence of ADRs in this study was lower. This may be primarily attributed to the study setting. Our study was population-based while most others were hospital-based. The hospitalized participants were likely to have more complex and serious diseases and were monitored more frequently, thus increasing the chances of discovering ADRs. Moreover, because the participants in our study were outpatients, mild or even transitory symptoms were occasionally not reported as the patients did not consider them relevant. ADRs due to anti-TB drugs are related to various factors. The principal determinants of such reactions are the dose and time of day at which the medication is administered, as well as patient ethnicity, age and nutritional status, together with the presence of preexisting diseases or dysfunctions, such as alcoholism, impaired liver function, impaired kidney function, drug interaction, and HIV co-infection [6], [13], [14], [27]. In addition, the incidence of ADRs is greatly influenced not only by the population sampled and the study setting, but also the methodology used to detect and classify the ADRs. These make comparison of reported rates between studies extremely difficult [28].

Similar to previous reports [10], [16], [29], most ADRs in our study occurred within the first two months after treatment. In this study, about 7% of ADRs were defined as serious, which was similar to previous reports [15]. Fortunately, most cases of ADRs (95%) had a good clinical outcome, which was in line with other studies [6], [7], [12]. A total of 593 (77.4%) patients with ADRs had extra clinic visits and resulted in a substantial increase in health care services.

### Impact of ADRs on Anti-TB Treatment

Our results showed that 7.6% (325/4304) of TB patients required modifying their anti-TB treatment due to ADRs. Schaberg T *et al* reported that the termination of isoniazid, rifampin or pyrazinamide because of severe side effects was necessary in 121 out of 519 patients (23%) [13]. An observation in routine treatment showed that 5.1% TB patients required modification of anti-TB treatment [8]. To our knowledge, minimal data exists concerning the impact of ADRs on anti-TB outcomes. Our results showed that patients with ADRs were at higher risk for unfavorable anti-TB outcomes. TB patients with unsuccessful anti-TB treatment outcomes were at higher risk to be multidrug-resistant and consequently had a lower probability of being cured [30]. According to the WHO report, one patient remaining in mycobacterium transmittable status could possibly infect 10 to15 more people in 12 months [31]. We also found that approximately 10.75% of all patients with positive smear result at the end of the intensive phase and 19.22% of all patients with unsuccessful outcomes may be attributed to ADRs due to DOTS therapy. Considering both that ADRs due to DOTS therapy was not rare and the large Chinese TB population, ADRs had a negative impact on the TB epidemic control in China.

The efficacy of DOTS therapy has been confirmed worldwide. The DOTS therapy success rate was 98.7% in this study. The world-wide average treatment success rate was 87% among new cases of sputum smear-positive pulmonary TB and it was 95% in China in 2010 [32]. However, DOTS therapy is a combination regimen of various drugs and is often difficult to evaluate the effectiveness or toxicity of a given drug. A thorough knowledge of pharmacokinetics and possible side effects of the drugs used in combination, as well as of the interactions among those drugs, will enable a clinician to treat patients with anti-TB drugs more safely and effectively [33]. In addition, it would be useful and practical to identify individuals who have risk factors for ADRs after initiating anti-TB treatment. It must be kept in mind that ADRs due to anti-TB drugs are not rare and they should be followed up by closer monitoring, especially in the first two months [12], [16], [29]. Guidelines for managing ADRs duo to DOTS therapy were developed in China in 2011 [34]. Fortunately, most ADRs can be managed well with the appropriate approach.

### Strength and Limitation

The main strength of this study was the standard longitudinal design, which represented one of the largest cohorts of TB patients receiving DOTS therapy. Although 184 patients were excluded, there were no statistically significant differences in terms of age and sex between the 4304 participants and the 184 patients (data not shown). The large sample size, diverse fields and intensive follow-up process enabled us to accurately determine the incidence of ADRs due to DOTS therapy and generalize the results to similar populations under certain conditions. We are aware of the fact that the incidence of ADRs in this study may be underestimated. The two routine tests may limit the ability of the study to detect some ADRs that were only laboratory documented. However, most ADRs due to anti-TB treatment showed symptoms and we believe the present data to be a realistic assessment. The classification of ADRs in this study was mainly based on the affected systems as suggested by experts, not exactly based on the Common Terminology Criteria for Adverse Events. It should be cautious to compare the incidence of ADRs due to DOTS therapy among similar studies. Another limitation was that lack of routine drug sensitivity testing, Hepatitis C co-infection, radiological extent of disease and whether homelessness or not were not recorded in this study and their impact on anti-TB outcomes was not adjusted for. The impact of ADRs on anti-TB outcomes may be overestimated. The prevalence of HIV was low (about 0.058%) in whole Chinese population [35] and the HIV status was not tested in this study.

### Conclusion

This study showed that 15.08% of TB patients who received DOTS therapy developed one or more ADRs. ADRs may result in an increase in health care services and affect the anti-TB treatment pattern. Patients with ADRs were more susceptible to develop unfavorable anti-TB outcomes. Given the incidence of ADRs and the size of the TB population in China, the negative impact of ADRs on anti-TB treatment would be substantial. This highlighted the importance of developing strategies to ameliorate ADRs both to improve the quality of patient care and to control TB safely.
